# Effect of salinity stress on the life history variables of *Branchipus schaefferi *Fisher, 1834 (Crustacea: Anostraca)

**DOI:** 10.1186/1746-1448-1-4

**Published:** 2005-07-04

**Authors:** SSS Sarma, Lynda Beladjal, S Nandini, Gerardo Cerón-Martínez, Karina Tavera-Briseño

**Affiliations:** 1 Laboratory of Aquatic Zoology, UMF, Division of Research and Postgraduate Studies, National Autonomous University of Mexico, Campus Iztacala, Av. de los Barrios S/N, Los Reyes, AP 314, CP 54090, Tlalnepantla, State of Mexico, Mexico; 2 Laboratory of Animal Ecology, Ghent University, Ledeganckstraat 35, B-9000 Gent, Belgium; 3 UIICSE, Division of Research and Postgraduate Studies, National Autonomous University of Mexico, Campus Iztacala, Av. de los Barrios S/N, Los Reyes, AP 314, CP 54090, Tlalnepantla, State of Mexico, Mexico

## Abstract

**Background:**

Freshwater anostracans inhabit ephemeral water bodies in which as the water level decreases due to evaporation the salt concentration increases. Thus, for most anostracans salinity becomes the major stress factor.

**Results:**

We tested five concentrations of NaCl (0 to 8 g/l) on the life table demography of *Branchipus schaefferi *fed *Chlorella *(alga). Age-specific survivorship curves of male and female *B. schaefferi *showed nearly a similar pattern in that increased salt concentration resulted in decreased survivorship. The age-specific reproduction (m_x_) of females showed several peaks of cyst production at 0 and 1 g/l salinity while in treatments containing salt at 4 or 8 g/l, there were fewer peaks. Average lifespan, life expectancy at birth, gross and net reproductive rates, generation time and the rate of population increase were all significantly influenced by the salt concentration in the medium. The highest value of net reproductive rate (970 cysts/female) was in treatments containing 0 g/l of salt, while the lowest was 13 cysts/female at 8 g/l. The rate of population increase (r) varied from 0.52 to 0.32 per day depending on the salt concentration in the medium.

**Conclusion:**

The low survival and offspring production of *B. schaefferi *at higher salinity levels suggests that this species is unlikely to colonize inland saline water bodies. Therefore, the temporary ponds in which it is found, proper conservative measures must be taken to protect this species.

## Background

Freshwater anostracans usually inhabit ephemeral waterbodies, which periodically dry, especially during summer months. This condition forces them to adapt to a) mostly one population cycle, b) produce cysts that resist desiccation and c) survive under changing ionic composition of the ambient medium [1]. The ionic composition of freshwater bodies is controlled by many factors. Among abiotic factors, temperature, through evaporation, plays an important role. Usually as the water level decreases due to evaporation, salt concentration increases. Therefore, both flora and fauna of such waterbodies must show some degree of salt tolerance in order to survive [2]. Knowledge on the pond salinity effects on the osmoregulation and conformation of anostracans is much limited. For example, *Branchinecta gigas *Lynch, 1937 and *B. mackini *Dexter, 1956 from saline lakes in Central Washington revealed that both these species have the capacity of hyperosmoregulation during low saline conditions and osmoconformation during high salinity conditions [3]. On the other hand, *Branchinella compacta *Linder, 1941 occurs in low saline conditions and has less tolerance to high salinity [4]. Thus, it remains unknown the tolerance capacities of many anostracan species.

Freshwater anostracans can survive during the drying period, yet their tolerance to increased salt levels is not well documented [1]. Mere survival under a given natural stress is not adequate for the continuation of a species; the reproductive output too is important. Thus, the reproductive strategies of freshwater anostracans reveal the vicissitudes of their habitat [5]. For most freshwater organisms including crustaceans, salinity is a major stress factor [6]. Increase in salinity leads to reduced survival, reduced reproductive output or both. For a given freshwater species, at salt concentrations below the median tolerance limits, reproduction is more drastically affected than survival or other variables such as swimming speed or the feeding rate [7].

The life history characteristics of crustaceans, in general, are best understood using life table demographic studies [8]. The variables more sensitive to stress are a) average lifespan, b) life expectancy at hatching, c) gross reproductive rate and d) net reproductive rates, e) generation time and f) the rate of population increase. However, not all variables are consistently sensitive to the same stress. Usually, the rate of population increase is thought to be more sensitive than the rest of the life history variables since it integrates both the mortality and natality [9]. Similar trends may be found in freshwater anostracans to salinity, but quantitative data are lacking for many genera except *Artemia *[10].

The aim of the present work was to evaluate the effect of different concentrations of sodium chloride on the life table demography of the freshwater anostracan *Branchipus schaefferi *Fisher, 1834.

## Results

### Survivorship curves

**Figure 1:**
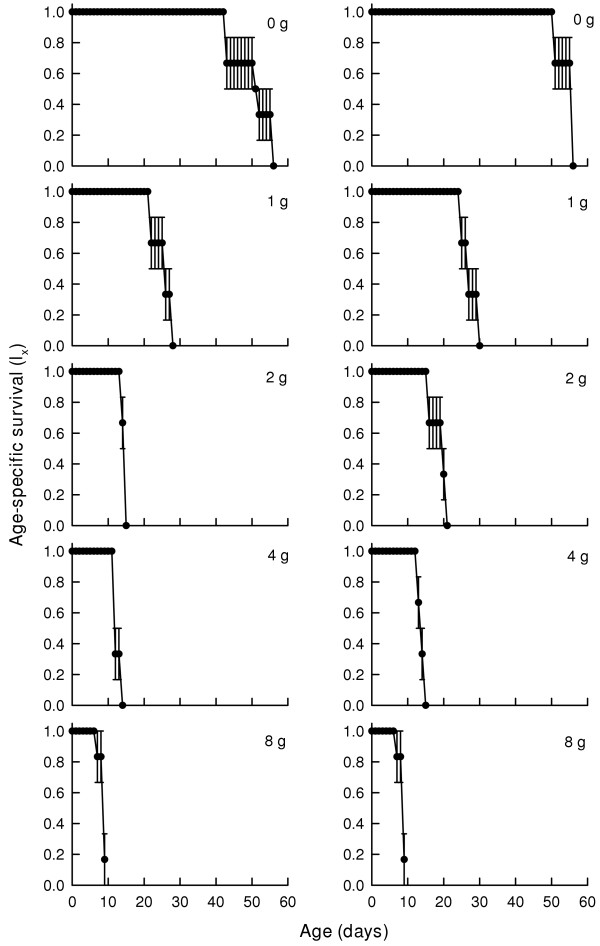
Age-specific surviorship (l_x_) curves of *B. schaefferi *subjected to different concentrations of NaCl. Column A: female; Column B: male. Values represent mean ± standard error based on 3 cohorts.

**Figure 2:**
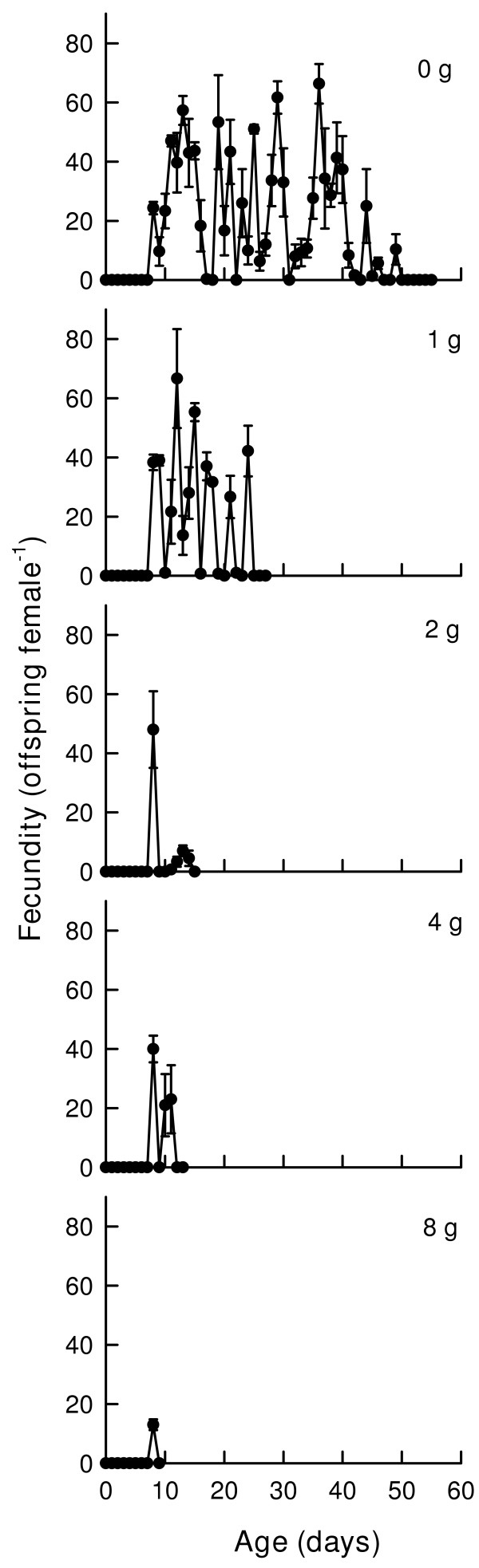
Age-specific fecundity (m_x_) curves of female *B. schaefferi *subjected to different concentrations of NaCl. Values represent mean ± standard error based on 3 cohorts.

Age-specific survivorship curves (Fig. 1) of female (A) and male (B) *B. schaefferi *showed a nearly similar pattern in relation to salt concentration (from 0 g to 8 g/l). At the highest salt concentration (8 g/l), females continued to live without mortality for the first week. The age-specific reproduction (m_x_) (Fig. 2) of females showed several peaks of cyst production at 0 and 1 g/l salinity while in treatments containing 4 or 8 g/l, there were fewer peaks.

### Life history variables

All the selected life history variables of *B. schaefferi *were significantly affected at even the lowest salinity increase (Table 1). Life expectancy at birth of males in 0 g/l salinity was slightly higher than females. However, in treatments containing a certain concentration of NaCl, this was nearly similar for both sexes. Also, regardless of sex, increased concentration of salt in the medium decreased the life expectancy. At the highest salt concentration, the life expectancy was nearly 1/6th of the controls (0 g/l). Gross and net reproductive rates also decreased with increasing salt level in the medium. The highest net reproductive rate (970 cysts/female) was in treatments containing 0 g/l salt, while the lowest (13 cysts/female) at 8 g/l.

**Table 1:** Selected life history variables of *Branchipus schaefferi *exposed to different concentrations of salt. For a given variable, treatments carrying same alphabet are not statistically significant (p > 0.05, Tukey's test)

Salt conc. (g/l)	Life Expectancy at birth (days)	Gross Reproductive rate (cysts / female)	Net reproductive rate (cysts / female)	Rate of pop. increase (r)
	**Female**			
0	49.5 ± 1. 9^a^	990.8 ± 47.0^a^	969.7 ± 37.4^a^	0.515 ± 0.004^a^
1	24.8 ± 0.8^b^	412.3 ± 14.6^b^	395.0 ± 20.9^b^	0.542 ± 0.002^b^
2	14.2 ± 0.2^c^	63.5 ± 6.9^c^	62.0 ± 8.1^c^	0.480 ± 0.003^c^
4	12.2 ± 0.3^c,d^	84.0 ± 24.1^c,d^	84.0 ± 24.1^c^	0.490 ± 0.02^c^
8	8.5 ± 0.1^d^	13.0 ± 1.8^d^	13.0 ± 1.8^c^	0.318 ± 0.01^c^
				
	**Male**			
0	53.8 ± 1.7^a^			
1	26.8 ± 1.4^b^			
2	18.5 ± 1.5^c^			
4	14.5 ± 0.2^d^			
8	8.3 ± 0.2^e^			

**Figure 3:**
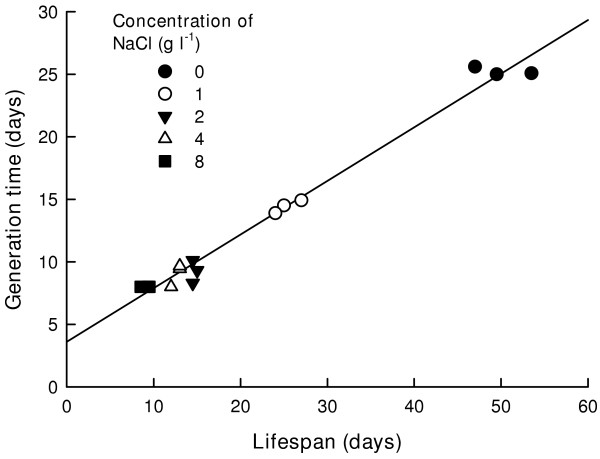
Relation between average lifespan and generation time in *B. schaefferi *under different concentration of NaCl. Plotted are the replicated data for each treatment.

The rate of population increase (r) varied from 0.52 to 0.32 depending on the salt concentration in the medium. As in the case of other survivorship variables, r decreased with an increase in NaCl in the medium. Generation time varied from 8 to 25 days, depending on the salt concentration. There was a positive relation between average lifespan and the generation time of *B. schaefferi *(Fig. 3).

Statistically, all the tested variables (survivorship variables: average lifespan, life expectancy at birth, reproductive variables: gross and net reproductive rates, generation time and the rate of population increase) were significantly influenced by the salt concentration in the medium (p < 0.001, one-way ANOVA). Tukey's tests revealed that all the above variables at 0 g/l salinity were significantly different (p < 0.05) from the treatments containing some quantity of NaCl. However, reproductive variables at 2, 4 and 8 g/l were not significantly different (p > 0.05).

## Discussion

Anostracans are particularly well suited for studying the impact of salt concentrations on the life history characteristics because they live in aquatic environments with a continually changing ionic composition [11]. Consequently, within its lifespan, an individual experiences different salinities, which in turn may affect the survival and reproductive performance [5].

Many species of freshwater anostracans feed on green algae [1]. *Branchipus schaefferi *was earlier cultured on the green agla *Scenedesmus *[12], which suggests that the use of algal diet was adequate for both survival and reproduction. This was evident in our study too, where in the absence of salt stress, *B. schaefferi *was able to survive and reproduce cysts.

Temporary waterbodies are characterized by opportunitistic species including anostracans. High reproductive output, short lifespan and wide diet breadth are some of the characteristics of crustacean species inhabiting temporary waters [2,5]. Many species of freshwater anostracans have a lifespan varying from about two weeks to several months [1]. In the present study, regardless of salt concentration, the maximum lifespan varied from 10 to 55 days. Compared to a previous study on the same species [13], the maximum lifespan in this study was slightly shorter (55 vs 77 days). However, as documented earlier, both males and females had nearly the same lifespan [13]. Depending on the environmental conditions, the age-specific life survivorship curves of anostracans may be type I (low initial mortality), type II (mortality rate independent of the age) or type III (heavy initial mortality) [14]. Usually under stressful conditions, the age-specific survivorship curves tend to be type III [15]. Increase in salt concentration in this study was a stress for *B. schaefferi *and therefore, with increasing NaCl level in the medium, there was steep fall in the survivorship in the initial age groups. Salt levels as low as 1 g/l caused about 80% mortality within 30 days for both males and females. At 8 g/l, both males and females lived for about a week without mortality and thereafter the survival rapidly declined.

Variable (saw tooth-like) cyst production is characteristic of crustaceans including anostracans that live more than 2 months [14,16], where offspring production is pulsed, i.e., after 5–10 day intervals neonate production peaks and during the interim period fewer eggs are produced. In *B. schaefferi *too, in treatments containing no salt or 1 g/l level, the offspring production was pulsed. With increased salinity in the medium fewer offspring were produced suggesting that salt levels beyond 2 g/l are highly stressful for *B. schaefferi*. The maximum cysts per brood was about 70. Reproduction was extremely low at 8 g/l. The number of cysts per brood for *Streptocephalus *may be as high as 900; however under inappropriate conditions this number may be as low as 1 cyst/brood [17]. In our study, the peak cyst production per brood was <20 at the highest salinity (8 g/l). When cultured on a diet of *Scenedesmus *at a density of 1 × 10^6 ^cells/ml, the maximum number of cysts per brood of *B. schaefferi *was 192 [13]. In the present study, this was much lower. This was probably due to the food density used. Based on dry weight [18], the quantity of algal diet used by Beladjal et al. [13] was nearly twice that used in this study. There is abundant evidence that increase in algal density increases the cyst production in anostracan species [1]. Generation time and rate of population increase observed for *B. schaefferi *are in broad agreement with those reported for freshwater anostracans [14].

Interrelationships exist among different life history variables of organisms. For example, body size and clutch size relation in crustaceans is generally positive and linearly or curvilinearly related [19]. Similarly generation time and lifespan are linearly related for many species of zooplankton such as rotifers [20], cladocerans [19], a few anostracans (e.g., *Streptocephalus mackini *Moore, 1966) [14] and now also in *B. schaefferi*.

The low tolerance capacity of *B. schaefferi *to NaCl as observed in the present study is also confirmed from field observations. For example, Maier et al. [11] have reported the occurrence of *B. schaefferi *in man-made freshwater bodies in Germany. However, with an increase in conductivity (higher than 300 μS/cm which is equivalent to <0.5 g/l), *B. schaefferi *was almost eliminated. There are also some differences with reference to the ionic composition of water in naturally drying ponds and NaCl used here [6]. However, species tolerant to other salts are also tolerant to NaCl or vice versa [21]. For example, *Branchinecta sandiegonensis *Fugate, 1993 and *Streptocephalus woottoni *Eng, Belk and Eriksen, 1990 which are generally found in dilute coastal vernal pools, are strong hyperregulators when external Na^+ ^levels are below 60 mmol^-1 ^or under conditions of alkalinities up to 0.8–1.0 g l^-1 ^[22]. Among anostracans, *Artemia *spp. are both hypo-and hyperosmotic regulators. For example, *Artemia franciscana *Kellog, 1906 showed little variations in haemolymph ion concentrations when the external salinity (as NaCl) was < 0.3 g l^-1 ^to supersaturated levels. However, this capacity has been reduced when exposed to low pH conditions [23]. Therefore it appears unlikely that *B. schaefferi *in natural ponds tolerates salinity levels higher than 8 g/l, especially under low pH conditions.

## Conclusion

*B. schaefferi *which inhabits temporary freshwater ponds tolerated only low NaCl levels. When raised on *Chlorella *at a density of 1 × 10^6 ^cells/ml under 0 g /l of NaCl concentration, both males and females had similar lifespan. However, when the salinity of the medium was increased from 0 to 8 g/l, both survival and reproduction were decreased. NaCl as low as 1 g/l caused negative influence on the life expectancy at birth, gross and net reproductive rates as well as the rate of population increase. Thus the low tolerance to salinity of this species suggests that it is unlikely to colonize inland saline waterbodies. Even in those freshwater ponds where it is found, anthropogenic factors leading to elevated salinity may eventually dislodge this species from its natural habitat [24]. In Germany *B. schaefferi *is considered as one of the most endangered crustacean species. Therefore, the temporary ponds in which it is found, proper conservative measures must be taken to protect this species.

## Methods

### Culture of test species

The original parental stock of *Branchipus schaefferi *was obtained from Boughzoul, Algeria and mass cultured using *Scenedesmus*. Cyst collection was done according to methods described previously [13]. Cysts were hatched following Ali et al. [17]. From the naupliar stage until the termination of the experiments, we used the single-celled alga *Chlorella vulgaris*. *C. vulgaris *(Strain CL-V-3, CICESE, Ensenada, Baja California, Mexico) was mass cultured using Bold's basal medium [25]. Based on a preliminary study, we selected 1.0 × 10^6 ^cells/ ml of *Chlorella *as the food density. Analytical grade sodium chloride was used for preparing different salinity levels. For cyst hatching, maintaining *B. schaefferi *in cultures or for experiments we used reconstituted moderately hard water (the EPA medium) [26] as the medium, which was prepared by dissolving 0.9 g of NaHCO_3_, 0.6 g of CaSO_4_, 0.6 g of MgSO_4 _and 0.04 g of KCl per litre of distilled water.

### Experimental design

Based on a preliminary study, five nominal concentrations of NaCl (0, 1, 2, 4 and 8 g/l) chosen. From a stock solution of 32 g/l, the desired concentrations were prepared through serial dilution using EPA medium. The effect of dilution on the algal density while preparing different salinity levels were considered and accordingly adjustments were done so as to obtain the final algal density of 1.0 × 10^6 ^cells /ml. The general test conditions were: fluorescent illumination (2000 Lux) continuous and diffused; temperature 23 ± 1°C, pH: 7.0–7.5, medium renewed completely after every 24 h.

Fifty nauplii were introduced into each of the 5 salt concentrations with an algal density of 1.0 × 10^6 ^cells/ml present in transparent test jars of 500 ml and the medium was renewed daily. After 10 days, by which time it was possible to distinguish the sex [13], the juveniles were used for conducting life table demographic studies. Into each of the 15 (5 salt concentrations X 3 replicates) test jars containing 100 ml medium we introduced one male and one female *B. schaefferi *(juveniles). Following initiation of the experiments, we counted the number of cysts from each test jar and the medium was renewed with appropriate salt level and algal density daily. If a male partner in a given test jar died then it was replaced by another one being grown simultaneously under similar conditions from the stock. Similarly if a female died then it was replaced by another female of similar test conditions, although the cyst count was not considered further [13]. The experiments were discontinued when every individual of the original pair died in each replicate. Based on the data collected, we derived age-specific survivorship (l_x_) and fecundity (m_x_) curves. The following formulae were used for obtaining life history variables [15]:

l_x _= Proportion of survivorship per day

m_x _= Proportion of offspring produced per female per day

where, T_x _= number of individuals per day

n_x _= number of living individuals at the initiation and the age × (days)

Rate of population increase, Euler-Lotka equation (solved iteratively and using jackknife method [27]):

where *r *= rate of population increase per day, *w *= age at maturity (days)

Differences in the data on life history variables obtained under different salt concentrations were statistically evaluated using analysis of variance (one-way ANOVA) and Tukey's tests [28].

## Competing interests

The author(s) declare that they have no competing interests.

## Authors' contributions

SSSS: Idea, design, some part of data collection and interpretation and write up; participated sufficiently to be an author of this manuscript.

LB: Culture, maintenance of organisms, some part of data analysis and interpretation; participated sufficiently to be an author of this manuscript.

SN: Some parts of data collection, analysis, interpretation and write up; participated sufficiently to be an author of this manuscript.

GCM: Some parts of data collection, interpretation and write up; participated sufficiently to be an author of this manuscript.

KTB: Some parts of data collection, interpretation and write up; participated sufficiently to be an author of this manuscript.
